# Clinicopathological Role of Serum-Derived Hyaluronan-Associated Protein (SHAP)-Hyaluronan Complex in Endometrial Cancer

**DOI:** 10.1155/2011/739150

**Published:** 2011-09-04

**Authors:** Hiromitsu Yabushita, Keita Iwasaki, Kouhei Kanyama, Yukihiko Obayashi, Lisheng Zhuo, Naoki Itano, Koji Kimata, Akihiko Wakatsuki

**Affiliations:** ^1^Department of Obstetrics and Gynecology, School of Medicine, Aichi Medical University, Nagakute-cho, Aichi 480-1195, Japan; ^2^Department of Obstetrics and Gynecology, Toyokawa City Hospital, Toyakawa, Aichi 422-8561, Japan; ^3^Research Complex for the Medicine Frontiers, School of Medicine, Aichi Medical University, Nagakute-cho, Aichi 480-1195, Japan; ^4^Department of Molecular Biosciences, Faculty of Life Sciences, Kyoto Sangyo University, Motoyama, Kamigamo, Kita-ku, Kyoto 603-8555, Japan

## Abstract

The role of hyaluronan (HA), serum-derived HA-associated protein (SHAP)-HA complex and hyaluronan synthase (HAS) in endometrial carcinomas was investigated. The relationship of metalloproteinase (MMP) and its inhibitor (TIMP) with HA and the SHAP-HA complex was also examined. The expression of HAS1 was related to the depth of myometrial invasion and lymph-vascular space involvement. The serum levels of HA, SHAP-HA complex, MMP-9, and TIMP-1 were increased in related with the depth of myometrial invasion, histological grade and lymph-vascular space involvement. They were also higher in the HAS1-positive group compared to -negative group. The serum concentrations of HA and SHAP-HA complex had a significant correlation with the MMP-9 and TIMP-1. The patients with elevated SHAP-HA complex had the shorter disease-free survival. The multivariate analysis revealed that the SHAP-HA complex was the independent variable for disease-free survival of endometrial cancer patients. In conclusion, the elevation of serum SHAP-HA complex depended on the HAS1 expression and the SHAP-HA complex is a useful marker to predict disease recurrence in endometrial cancer patients. The SHAP-HA complex may promote the lymph-vascular space involvement and the synthesis and activation of MMP-9 and TIMP-1 in the progression of endometrial cancer.

## 1. Introduction

The incidence rate for endometrial cancer has been increasing in Japan [1]. Clinicopathological studies show that poor prognosis is related to cervical invasion of malignant cells, deep myometrial invasion of malignant cells, lymph node metastasis, and lymph-vascular space involvement of malignant cells [2, 3]. The multistage process of tumor invasion and metastasis depends on several mechanisms, including the stimulation of cell growth by growth factors, destruction of the extracellular matrix by proteolytic enzymes, neovascularization due to the presence of angiogenic factors, and cell to cell or stroma adhesion regulated by cell adhesion molecules.

Hyaluronan (HA) is an extracellular polysaccharide typically present in the extracellular matrix of some epithelial and neural tissues. HA is particularly abundant in connective tissues. HA controls cell migration, differentiation, and proliferation, thereby influencing tissue morphogenesis, wound healing, and tumor growth [4, 5]. HA levels correlate with the invasiveness and metastatic capacity of tumor cells [6]. Increased HA concentrations may help invasion by providing a less dense matrix for cancer cells [7], stimulating cancer cell motility, and forming an immunoprotective coat for cancer cells [8].

Three mammalian hyaluronan synthase (HAS) genes, HAS1, HAS2, and HAS3, are involved in hyaluronan biosynthesis [9], and their expressions vary depending upon tissue-, age- and pathophysiological differences and are important to investigate the biological function of hyaluronan, especially in tumor cell malignancy, because the overexpression of HAS makes tumor cells more aggressive with significant differences in the malignant activity among the three HAS genes [10]. The three isoforms of HAS have been shown to synthesize distinct types of hyaluronans with different structural characteristics and functions [9].

Associations with various HA-binding proteins, including proteoglycans, result in tremendously diverse physiological functions for HA. Extracellular matrix containing HA as a major component, called HA-rich matrix, plays important roles in regulating cellular behavior in a variety of physiological and pathological processes via cell-surface HA receptors, such as CD44 and receptor for hyaluronan-mediated motility [11].

SHAP was originally discovered as the complex with HA in HA-rich matrix from cultured mouse dermal fibroblasts and was found to be derived from serum supplemented to culture media, and thereby named as serum-derived HA-associated proteins (SHAPs) [12, 13]. Then, SHAP was found to correspond to the heavy chains of plasma inter-*α*-trypsin inhibitor (ITI) family molecules and be covalently bound to HA via a unique ester bond [12–14]. ITI family molecules are synthesized by hepatocytes and secreted into the blood at high concentrations [15]. The heavy chains of these molecules are derived from 3 different genes, and either 1 or 2 of the chains are covalently bound to the light chain, bikunin, to form ITI family members such as ITI, pre-*α*-trypsin inhibitor, and inter-*α*-trypsin-like inhibitor [16]. During the formation of SHAP-HA complexes, HA is substituted for the chondroitin sulfate chain of bikunin, accompanied by the release of bikunin [12, 17]. Released bikunin is excreted in urine as urinary trypsin inhibitor (UTI). 

Matrix metalloproteases (MMPs) play a crucial role in tissue remodeling in a variety of physiological and pathological processes, similar to the roles of HA. In malignant tumor, HA may possibly influence the expression of MMP-9 as well as the conversion of the inactive pro-forms to active forms [18, 19].

We previously reported the role of HA and HAS1 in endometrial cancer [20]. The expression of HAS1 and serum HA level were related to the depth of myometrial invasion, histological grade, and lymph-vascular space involvement. Serum HA level was higher in the HAS1-positive group than in the HAS1-negative group. The goal of the present study was to determine if the levels of HA, the SHAP-HA complex, UTI, and the immunohistochemical expression of HAS correlate with the clinicopathological manifestations and clinical outcome of endometrial carcinoma. The causal relationships of the serum levels of HA and the SHAP-HA complex with those of metalloproteinase and its inhibitor in serum were also examined and discussed.

## 2. Materials and Methods

### 2.1. Clinical Samples

Formalin-fixed, paraffin-embedded tissue, serum, and urine were obtained from 50 patients with endometrioid adenocarcinoma of the endometrium. All patients attended the gynecology clinic at Aichi Medical University Hospital and were diagnosed as stage I. Of them, nine cases who did not undergo lymphadenectomy had no finding suggesting the lymph node metastasis in a presurgical CT scan examination. The surgical specimens were evaluated by more than one gynecologic pathologists, and they also evaluated the immunohistochemical examination. Serum and urine were also obtained from 50 postmenopausal donors with the negative endometrial cytology. The mean age of endometrial cancer patients was 57.24 ± 9.28 years, and that of healthy women was 54.52 ± 6.34 years observed in healthy women, which was almost the same as that of the patients (*P* = 0.09). This study was approved by the regional ethics committee of Aichi Medical University, School of Medicine. Written informed consent was obtained from all participants prior to study enrollment.

### 2.2. Immunohistochemistry

The polyclonal antibodies against HAS1, HAS2, and HAS3 were raised in rabbits by subcutaneous injection of the following synthetic peptides; VRRLCRRRSGGTRVGV, corresponding to amino acids 568–582 of HAS1; CGRRKKGQQYDMVLDV, corresponding to the amino acids 537–552 of HAS2; CGKKPEQYSLAFAEV, corresponding to amino acids 541–555 of HAS3, which had been coupled to keyhole limpet hemocyanin. The specificity of the purified antibodies has been confirmed [10, 21]. Immunostaining was performed by the avidin-biotin-peroxidase complex method using a VECTASTAIN ABC kit Elite (Vector Laboratories Inc., Burlingame, Calif, USA). The prepared 3 *μ*m sections were deparaffinized and rehydrated. After treatment with 0.25% trypsin solution at 37°C for 10 minutes, the sections were incubated at 4°C overnight with the antibodies against HAS1, HAS2 and HAS3. The chemogen used was 3,3′-diaminobenzidine tetrahydrochloride (Dojindo, Kumamoto, Japan). Sections were counterstained with hematoxylin for microscopic examination. Sections were defined as having positive expression when >90% of tumor cells were intensely stained.

### 2.3. Measurement of Serum Concentrations for HA, SHAP-HA Complex, MMP-2, MMP-9 and TIMP-1, and Urine Concentration of UTI

Concentrations of HA in serum were measured using an inhibitory enzyme-linked immunosorbent assay (ELISA) (Seikagaku Corp., Tokyo, Japan) [22]. Briefly, HA-conjugated bovine serum albumin (BSA) plates were washed 3 times with phosphate-buffered saline (PBS) containing 0.1% Tween 20 (PBS-T). Then, 50 *μ*L sample (diluted 1 : 5~10 with PBS-T) and 50 *μ*L biotinylated HABP (0.5 *μ*g/mL in 1% BSA/PBS-T) were applied to each well, and plates were incubated at 37°C for 1 h. After washing with PBS-T, 50 *μ*L horseradish peroxidase-streptavidin (1 : 500) was added to each well, and plates were further incubated at 37°C for 1 h. Color development was achieved by incubating with 50 *μ*L of tetramethylbenzidine (TMB) solution at 37°C for 10 min; then, the reaction was stopped by adding 50 *μ*L 1 M HCl. Absorbance at 450/630 nm (absorbance at 450 nm-absorbance at 630 nm) was measured with an immuno Mini NI-2300 spectrophotometer. Assays were performed in triplicate.

The levels of the SHAP-HA complex in sera were determined by measuring the amount of SHAPs bound to HA using a sandwich ELISA [22]. Microtiter plates were coated with HABP (4 *μ*g/mL in 0.1 M sodium carbonate buffer; pH 9.5) at 4°C for 15 h. Wells were washed twice with 200 *μ*L PBS, followed by blocking with 200 *μ*L of 3% BSA in PBS-T at room temperature for 1 h. After washing the wells 3 times with 200 *μ*L PBS-T, 50 *μ*L sample (serum diluted 1 : 5~10 with 1% BSA/PBS-T) was added to each well. Then, plates were incubated at 37°C for 1 h. After washing, 25 *μ*L of rabbit antihuman ITI antibody (diluted 1 : 3000 with 1% BSA/PBS-T) and 25 *μ*L of HRP-conjugated goat antirabbit immunoglobulins antibody (diluted 1 : 3000 with 1% BSA/PBS-T) were added to each well and incubated at 37°C for 1 h. Wells were washed 3 times then were incubated with 50 *μ*L of TMB solution at 37°C for 10 min. The reaction was stopped by the addition of 50 *μ*L 1 M HCl, and absorbance at 450/650 nm was measured. Assays were performed in triplicate.

The urine levels of UTI were measured using an inhibitory ELISA [22]. Microtiter plates were coated with UTI (2 *μ*g/mL in 0.1 M sodium carbonate buffer; pH 9.5) at 4°C overnight and then blocked with 3% BSA as above. The plates were washed 3 times with PBS-T, then 50 *μ*L sample (urine diluted 1 : 5~10 with PBS) and 50 *μ*L anti-UTI antibodies were applied to each well and incubated at 37°C for 1 h. Wells were washed, and 100 *μ*L HRP-conjugated goat anti-rabbit antibody was added to each well. Plates were then further incubated at 37°C for 1 h. After washing with PBS-T, color development was achieved by incubation with 50 *μ*L TMB solution at 37°C for 10 min, then the reaction was stopped using 50 *μ*L 1 M HCl. Absorbance at 450/650 nm was measured. Assays were performed in triplicate. 

Concentrations of MMP-2, MMP-9, and TIPM-1 in sera were measured with a one-step sandwich ELISA kit for each (Fuji Chemical Co., Toyama, Japan) as reported previously [23–25]. A serum (10 *μ*L) was mixed with 100 *μ*L of 50 *μ*g/L each antibody conjugated with HRP in 10 mM sodium phosphate buffer (pH 7.0) containing 10 g/L BSA, 10 nM EDTA, and 0.1 M NaCl. A 100 *μ*L aliquot of the mixture was transferred to microplate well previously coated with each antibody. The plate was incubated for 60 min at room temperature and then washed 3 times with PBS. Color development was achieved by incubation with 100 *μ*L of citric acid-sodium phosphate buffer containing *o-*phenylenediamine and hydrogen peroxide at room temperature for 20 min. The reaction was stopped with 100 *μ*L 1 M HCl, and the absorbance at 492 nm was measured. Assays were performed in triplicate.

### 2.4. Statistical Analysis

The statistical significance of differences among different categories of expression was analyzed using the unpaired *t*-test, the chi-square test, and two-way ANOVA. Pearson correlation coefficient was used to test for significant relationships. Disease-free survival was analyzed by the Kaplan-Meier method and log-rank test, and the potential significance of plural prognostic factors for disease-free survival was analyzed by Cox proportional hazard model. *P* values less than 0.05 were considered statistically significant.

## 3. Results

### 3.1. HAS, HA, SHAP-HA Complex, UTI, MMP-2, MMP-9, and TIMP-1 in Relation to the Clinicopathological Manifestations

**Figure 1:**
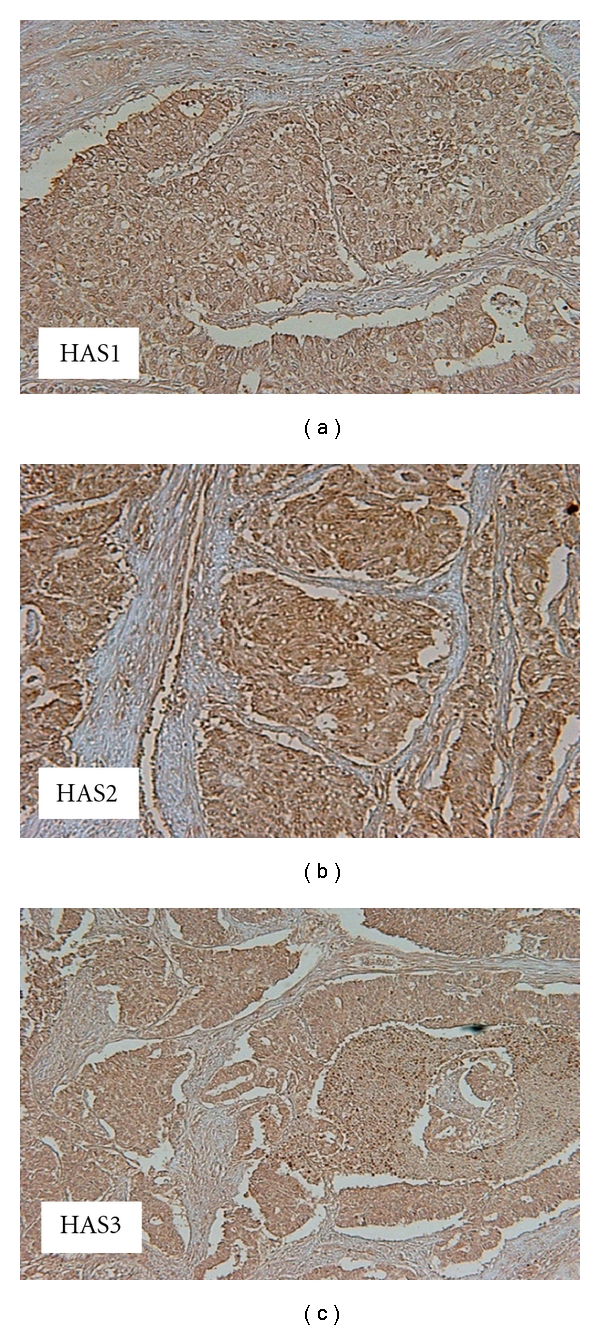
Immunohistochemical visualization of HAS1, HAS2, and HAS3 in endometrioid adenocarcinoma of the uterine corpus (×400).

In endometrioid adenocarcinoma of uterine corpus, the HAS1, HAS2, and HAS3 were expressed in tumor cells as our previous report (Figure 1) [20]. Of the 50 endometrial cancer cases, the positive immunohistochemical expression was found in 29 cases for HAS1, 33 cases for HAS2, and 29 cases for HAS3. The expression of HAS1 was related to the depth of myometrial invasion and lymph-vascular space involvement although it had no relationship with the histological grade. On the other hand, the expression of HAS2 and HAS3 had no relationship with the depth of myometrial invasion, histological grade, and lymph-vascular space involvement (Table 1). The serum levels of HA, the SHAP-HA complex, MMP-9, TIMP-1, and UTI were higher in the endometrial cancer group than in the control group, while the serum MMP-2 levels had no difference between both groups (Table 2). The serum levels of HA, the SHAP-HA complex, MMP-9, and TIMP-1 were significantly increased in the patients with the deeper myometrial invasion (Table 3). The urine levels of UTI and serum levels of HA, the SHAP-HA, complex and TIMP-1 were higher in the patients with the poorly differentiated (G3) tumor, compared with the moderately differentiated (G2) tumor and well-differentiated (G1) tumor. The serum levels of HA, the SHAP-HA complex, MMP-9, TIMP-1, and the urine level of UTI were significantly increased in the patients with the lymph-vascular space involvement (Table 3).

### 3.2. Relationship of Immunohistochemical Expressions of HAS Isoforms with Serum Levels of HA, the SHAP-HA Complex, MMP-2, MMP-9 and TIMP-1, and Urine Level of UTI

The serum levels of the SHAP-HA complex, MMP-9, and TIMP-1 were higher in the HAS1-positive group than in the HAS1-negative group. However, the expression of HAS2 and HAS3 was unrelated to them (Table 4). The serum levels of HA and the SHAP-HA complex had a significant correlation with the MMP-9, and TIMP-1 in endometrial cancer patients (Table 5).

### 3.3. HAS, HA, SHAP-HA Complex, UTI, MMP-2, MMP-9, and TIMP-1 Levels in Relation to the Disease-Free Survival and Overall Survival

**Figure 2:**
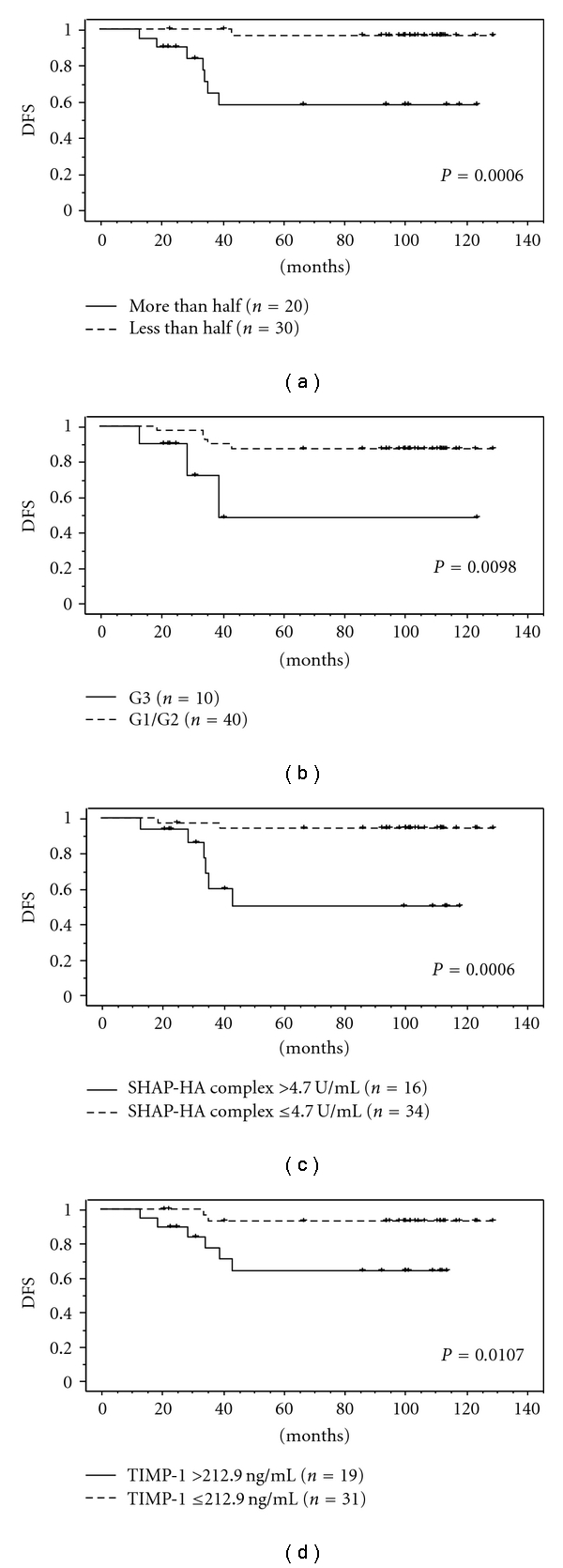
Disease-free survival in endometrial cancer patients was analyzed by the Kaplan-Meier method and log-rank test. The patients with the deeper myometrial invasion, poorly differentiated tumor, elevated serum level of the SHAP-HA complex, and elevated serum level of TIMP-1 had a significantly shorter disease-free survival. The maximal normal level in serum of the SHAP-HA complex in serum was determined as 4.7 U/mL for the SHAP-HA complex and as 212.9 ng/mL for TIMP-1, which were a value of the mean + 2SD in 50 healthy women.

**Figure 3:**
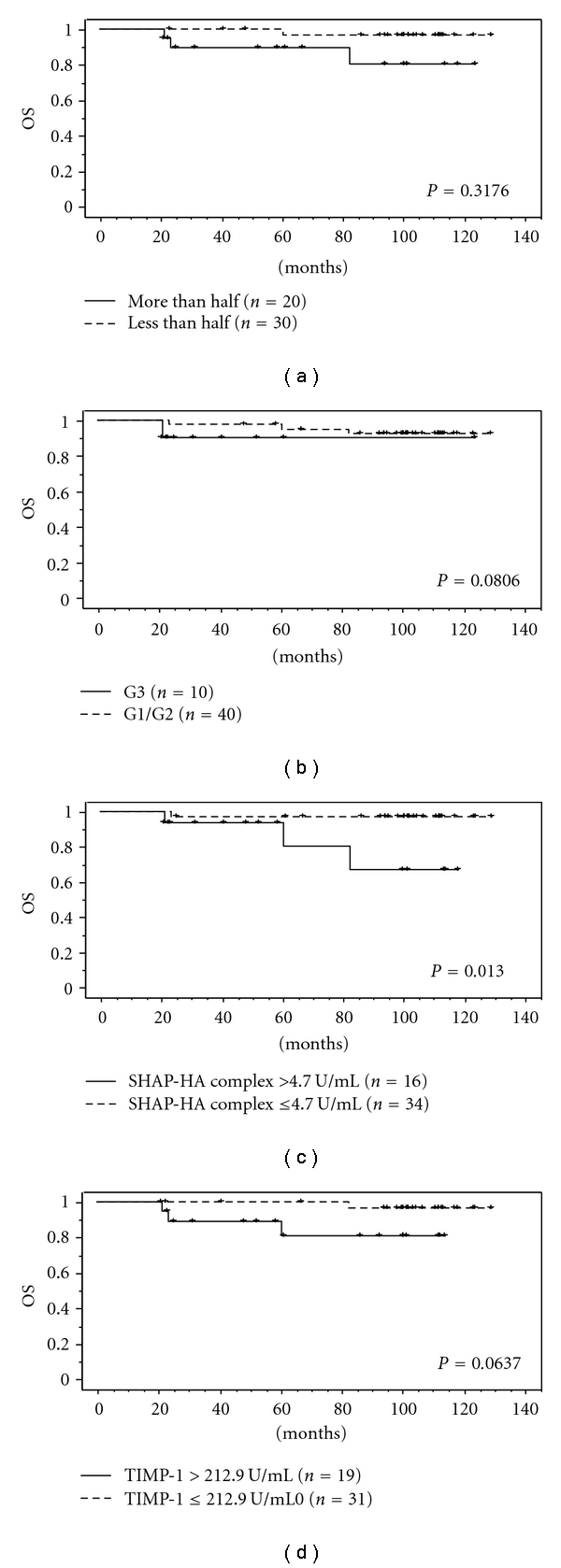
Overall survival in endometrial cancer patients was analyzed by the Kaplan-Meier method and log-rank test. The patients with elevated serum level of the SHAP-HA complex had a significantly shorter overall survival.

The Kaplan-Meier analysis revealed that the patients with elevated serum levels of the SHAP-HA complex and TIMP-1 had a shorter disease-free survival compared with those with normal levels of them. Also, the Kaplan-Maier analysis revealed that patients with the deeper myometrial invasion and poorly differentiated tumor had a significantly shorter disease-free survival (Figure 2). The multivariate analysis revealed that the SHAP-HA complex was the significant independent variable for the disease-free survival of endometrial cancer patients (Table 6). The Kaplan-Meier analysis revealed that the patients with elevated serum level of the SHAP-HA complex had a significantly shorter overall survival although no significant difference was found in relation with the serum TIMP-1 levels, depth of myometrial invasion, and histological grade (Figure 3). However, no significant variable was observed in the multivariate analysis for overall survival (Table 7).

## 4. Discussion

The multistage process of tumor invasion and metastasis depends on several mechanisms, including the stimulation of cell growth by growth factors, destruction of the extracellular matrix by proteolytic enzymes [26], neovascularization due to the presence of angiogenic factors [27, 28], and cell-to-cell or stromal cell interaction regulated by cell adhesion molecules and pericellular matrix molecules.

Significantly increased levels of HA are often associated with certain types of human tumors, and the levels of HA in the sera of some cancer patients are significantly higher than those of normal individuals [7, 29, 30]. Although increased HA synthesis is not a universal characteristic of tumors, there seems to be an overall tendency for transformed cells to exhibit higher levels of HA production [31]. In addition, a close relationship has been demonstrated between HA production and malignant phenotypes, such as invasiveness [32]. Our group and another group found that highly metastatic cell lines release more HA into culture medium than less metastatic variants [6]. Furthermore, Zhang et al. [33] reported that HA on the surface of tumor cells is correlated with metastatic behavior.

HA has either directly or indirectly been implicated in cell adhesion, motility, growth, and differentiation [34]. HA-binding proteins regulate these cellular behaviors by interacting with HA and forming the HA pericellular matrix [35]. Increased matrix deposition of HA may favor tumor growth and invasion by increasing tissue hydration and providing a suitable environment for cell migration analogous to embryonic cell movement. Other mechanisms may also help tumor growth and invasion. For example, the HA pericellular coat may reduce the access and attack of immune cells to tumor cells [36]. Tumor cells are surrounded by a thick pericellular coat that is sensitive to hyaluronidase. The removal of this coat may allow lymphocytes to exert their cytolytic effect on tumor cells. Additionally, partially degraded HA fragments promote angiogenesis, an important host contribution to tumor cell viability [37].

Our previous study [38] demonstrates that HA synthesized by HAS1 is associated with tumor neovascularization and predicts tumor aggressiveness and patient survival in ovarian cancer. Yamada et al. [39] reported that elevated transcription levels of the HAS1 gene correlated with poor prognosis of human colon cancer. Anttila et al. [40] reported that elevated levels of stromal HA indicate that the tumor is aggressive and predict poor disease outcome in ovarian cancer patients, especially those with serous tumors. Kayastha et al. [41] reported that CD44 expression is associated with the spread of ovarian cancer and is an independent predictor of survival. HA contributes to tumor growth, invasion, and metastasis through cell proliferation, movement, adhesion, and angiogenesis. It is possible that these functions of HA depend on the overexpression of the HAS genes.

In our previous report [38] and the present study, the serum levels of HA and the SHAP-HA complex were both higher in the endometrial cancer group than in the healthy control group, and the increased synthesis of HA in endometrial cancer tissue appeared to depend on the high expression of HAS1. This finding suggests that increased HA reacts with ITI, resulting in the increased levels of the SHAP-HA complex. In addition, the serum levels of HA and the SHAP-HA complex in those cancer group were found to be increased in accordance with the depth of myometrial invasion and lymph-vascular space involvement [38], indicating that the increased levels of HA and the SHAP-HA complex are related to tumor invasion and metastasis.

The present study has also demonstrated that the serum levels of MMP-9 and TIMP-1 were significantly higher in the endometrial cancer group than in the healthy control group, and serum MMP-9 was elevated according to the depth of myometrial invasion and lymph-vascular space involvement. There are some reports suggesting that hyaluronan upregulates MMP-9 and TIMP-1 expressions via CD44 signaling [42, 43]. Peng et al. [44] have shown that, in culture of human breast carcinoma cells, antibody-mediated CD44 cross-linking resulted in the colocalization of CD44 and MMP-9, which may augment the level of MMP-9 in the membrane, possibly implicated in the increased capacity of tumor invasion and metastasis. We have shown that SHAP appears to potentiate the interaction resulting in the increased avidity of HA to CD44 and have suggested that SHAP could function in activating CD44 signaling by increasing the interaction with HA [45]. It is likely therefore that the high serum levels of MMP-9 may be consequential reflection of the increased levels of the SHAP-HA complex in endometrial cancer, which explains the present observation that the elevated levels of SHAP-HA complex had a shorter disease-free survival time compared to those patients with normal levels of SHAP-HA complex.

 MMPs are known to be inhibited by naturally occurring TIMPs [46]. Thus, TIMPs are also important factors to regulate cancer cell invasion and metastasis. For one example, Runx3 has been shown to suppress gastric cancer metastasis through the inactivation of MMP-9 by the upregulation of TIMP-1 [47]. However, TIMPs were found to have multiple functions [48]. It not only regulates activity of MMPs as their natural tissue inhibitors as described above, but also stimulates tumor growth and malignant transformation. For example, tissue levels of TIMPs in gastric adenocarcinoma [49] and their serum levels in colorectal cancer [50] are reported to reflect roles in predicting the aggressive behaviors of those cancer cells, together with those of MMP-9, suggesting that MMP-9 expression is an independent diagnostic valuable for tumor malignancy. Or, the higher levels of TIMPs could reflect some physiological mechanisms of TIMPs expressions to regulate MMPs expressions and activities. Considering those multi- and variable functions of TIMPs, it is reasonable that the present analysis has revealed that the SHAP-HA complex and TIMP-1 were a significant independent variable predicting a shorter disease-free survival.

## 5. Conclusion

The elevation of serum SHAP-HA complex depended on the HAS1 expression and the SHAP-HA complex is a useful marker to predict disease recurrence in endometrial cancer patients. Because the levels of the SHAP-HA complex showed a significant positive correlation with the levels of MMP-9 and TIMP-1, the SHAP-HA complex may promote the lymph-vascular space involvement and the synthesis and activation of MMP-9 and TIMP-1 in the progression of endometrial cancer.

## Figures and Tables

**Table 1:** Expression of HAS1, HAS2, and HAS3 in relation to the depth of myometrial invasion, histological grade, and lymph-vascular space involvement in patients with endometrial cancer.

		HAS1 expression		HAS2 expression		HAS3 expression	
		Negative	Positive	Negative	Positive	Negative	Positive
	*n*	(*N* = 21)	(*N* = 29)	(*N* = 18)	(*N* = 32)	(*N* = 21)	(*N* = 29)
Myometrial invasion							
Less than half	30	16	14	11	19	14	16
More than half	20	5	15	7	13	7	13
		*χ* ^2^ = 3.96, *P* = 0.0467		*P* > 0.9999		*P* = 0.5602	

Histological grade							
G1/G2	40	19	21	16	24	15	25
G3	10	2	8	2	8	6	4
		*P* = 0.1604		*P* = 0.2947		*P* = 0.2859	

Lymph-vascular space involvement							
Absent	28	16	12	10	18	12	16
Present	22	5	17	8	14	9	13
		*χ* ^2^ = 5.99, *P* = 0.0213		*P* > 0.9999		*P* = 0.8898	

**Table 2:** Serum HA, SHAP-HA complex, MMP-2, MMP-9, TIMP-1, and UTI in 50 endometrial cancer patients and 50 healthy women.

	*n*	HA (ng/mL)	SHAP-HA complex (U/mL)	UTI (*μ*g/mL)	MMP-2 (ng/mL)	MMP-9 (ng/mL)	TIMP-1 (ng/mL)
Healthy control	50	52.74 ± 23.87	1.884 ± 1.418	1.930 ± 1.47	707.84 ± 124.20	277.76 ± 117.68	154.16 ± 29.37
Endometrial cancer	50	368.23 ± 233.28	3.294 ± 2.209	7.004 ± 15.044	703.94 ± 182.54	736.40 ± 524.24	223.80 ± 100.79
		*P* < 0.0001	*P* = 0.0002	*P* = 0.0197	*P* = 0.9009	*P* < 0.0001	*P* < 0.0001

Values are shown as mean ± SD.

**Table 3:** Serum HA, SHAP-HA complex, MMP-2, MMP-9, TIMP-1, and UTI in relation to the depth of myometrial invasion, histological grade, and lymph-vascular space involvement in patients with endometrial cancer.

	*n*	HA (ng/mL)	SHAP-HA complex (U/mL)	UTI (*μ*g/mL)	MMP-2 (ng/mL)	MMP-9 (ng/mL)	TIMP-1 (ng/mL)
Myometrial invasion							
Less than half	30	236.94 ± 145.14	2.151 ± 2.030	5.737 ± 13.679	720.03 ± 197.67	598.90 ± 439.17	199.27 ± 81.09
More than half	20	565.18 ± 200.37	5.009 ± 3.888	8.905 ± 17.080	679.80 ± 158.96	942.65 ± 582.89	260.60 ± 117.41
		*P* < 0.0001	*P* = 0.0014	*P* = 0.4713	*P* = 0.4508	*P* = 0.0215	*P* = 0.0336

Histological grade							
G1/G2	40	295.39 ± 180.95	2.443 ± 2.397	4.422 ± 11.565	699.50 ± 186.84	694.58 ± 504.76	209.40 ± 92.94
G3	10	659.63 ± 191.00	6.698 ± 3.883	17.330 ± 22.462	721.70 ± 172.34	903.70 ± 594.45	281.40 ± 115.21
		*P* < 0.0001	*P* < 0.0001	*P* = 0.0136	*P* = 0.7347	*P* = 0.2635	*P* = 0.0421

Lymph-vascular space involvement							
Absent	28	247.75 ± 147.98	2.340 ± 2.838	2.107 ± 1.767	682.32 ± 185.10	575.71 ± 328.58	179.75 ± 36.44
Present	22	521.57 ± 234.18	4.508 ± 3.304	13.236 ± 21.246	731.46 ± 179.66	940.91 ± 651.32	279.86 ± 126.95
		*P* < 0.0001	*P* = 0.0161	*P* = 0.0080	*P* = 0.3500	*P* = 0.0129	*P* = 0.0002

Values are shown as mean ± SD.

**Table 4:** Serum HA, SHAP-HA complex, MMP-2, MMP-9, TIMP-1, and UTI in relation to the expression of HAS1, HAS2, and HAS3 in patients with endometrial cancer.

	*n*	Hyaluronan (ng/mL)	SHAP-HA complex (U/mL)	UTI (*μ*g/mL)	MMP-2 (ng/mL)	MMP-9 (ng/mL)	TIMP-1 (ng/mL)
HAS1 expression							
Negative	21	298.22 ± 154.04	1.168 ± 0.917	2.390 ± 2.047	719.10 ± 207.79	504.76 ± 205.00	183.38 ± 67.60
Positive	29	418.93 ± 268.23	4.833 ± 3.403	10.345 ± 19.119	692.97 ± 164.84	904.14 ± 617.70	253.07 ± 111.36
		*P* = 0.0705	*P* < 0.0001	*P* = 0.0643	*P* = 0.6223	*P* = 0.0065	*P* = 0.0142

HAS2 expression							
Negative	18	349.8 ± 216.78	2.851 ± 2.624	3.756 ± 5.973	734.22 ± 212.04	787.28 ± 525.09	244.44 ± 120.89
Positive	32	378.6 ± 244.82	3.543 ± 3.511	8.831 ± 18.127	686.91 ± 164.86	707.78 ± 525.09	212.19 ± 87.5
		*P* = 0.6796	*P* = 0.4694	*P* = 0.2563	*P* = 0.3845	*P* = 0.6118	*P* = 0.2819

HAS3 expression							
Negative	21	344.49 ± 242.18	3.950 ± 3.684	8.010 ± 16.122	744.57 ± 157.21	798.76 ± 625.46	252.67 ± 122.38
Positive	29	385.43 ± 229.38	2.819 ± 2.778	6.276 ± 14.460	674.52 ± 196.27	691.24 ± 443.27	202.90 ± 77.48
		*P* = 0.5061	*P* = 0.2226	*P* = 0.6919	*P* = 0.1832	*P* = 0.4798	*P* = 0.0848

Values are shown as mean ± SD.

**Table 5:** Correlationship between the serum HA, SHAP-HA complex, MMP-2, MMP-9, TIMP-1, and UTI in patients with endometrial cancer.

Variables	*r*	*P* value
HA and SHAP-HA complex	0.466	0.0005
HA and UTI	0.306	0.0305
HA and MMP-2	0.007	0.9635
HA and MMP-9	0.345	0.0137
HA and TIPM-1	0.364	0.0089
SHAP-HA complex and UTI	0.235	0.1004
SHAP-HA complex and MMP-2	0.066	0.6523
SHAP-HA complex and MMP-9	0.455	0.0008
SHAP-HA complex and TIMP-1	0.396	0.0041
UTI and MMP-2	−0.061	0.6739
UTI and MMP-9	0.071	0.6242
UTI and TIMP-1	0.255	0.0736
MMP-2 and MMP-9	0.032	0.827
MMP-2 and TIMP-1	0.386	0.0053
MMP-9 and TIMP-1	0.677	<0.0001

**Table 6:** Hazard ratios for disease-free survival with 95% CIs adjusted for HAS, HA, SHAP-HA complex, UTI, MMP-2, MMP-9, TIMP-1, and the clinicopathological manifestations in patients with endometrial cancer.

Variables	Hazard ratio	95% CI	*P* value
Age	1.036	0.866–1.238	0.7020
Myometrial invasion (more than half)	79.594	0.194–3261.041	0.1155
Histological grade (G3)	3.407	0.146–79.578	0.4458
Lymph-vascular space involvement	0.892	0.010–77.881	0.9602
HAS1 expression	0.213	0.002–23.731	0.5203
HAS2 expression	0.273	0.010–7.258	0.4380
HAS3 expression	0.132	0.002–7.961	0.3325
HA	0.990	0.973–1.007	0.2440
SHAP-HA complex	1.773	1.010–3.111	0.0461
UTI	1.084	0.981–1.198	0.1146
MMP-2	0.993	0.976–1.011	0.4693
MMP-9	1.000	0.998–1.002	0.9204
TIMP-1	1.008	0.984–1.034	0.5074

**Table 7:** Hazard ratios for overall survival with 95%CIs adjusted for HAS, HA, SHAP-HA complex, UTI, MMP-2, MMP-9, TIMP-1, and the clinicopathological manifestations in patients with endometrial cancer.

Variables	Hazard ratio	95% CI	*P* value
Age	0.848	0.583–1.232	0.3859
Myometrial invasion (more than half)	3.846	0.027–544.845	0.5940
Histological grade (G3)	0.007	<0.001–7458.166	0.4799
Lymph-vascular space involvement	0.002	<0.001–175.169	0.2913
HAS1 expression	—	—	—
HAS2 expression	—	—	—
HAS3 expression	—	—	—
HA	1.003	0.984–1.023	0.7456
SHAP-HA complex	3.417	0.820–12.075	0.0947
UTI	0.297	0.055–1.610	0.1591
MMP-2	1.010	0.992–1.029	0.2790
MMP-9	1.004	0.996–1.013	0.3126
TIMP-1	—	—	—
